# Mixed-methods feasibility study of a community-based model to improve equity and efficiency in dementia research participation: protocol for ACCESS D

**DOI:** 10.1136/bmjopen-2026-118119

**Published:** 2026-07-13

**Authors:** Patricia Fuller, Andrew Claxton, Helen Pocock, Samantha Jane Williams, Nicola Claxton, Amanda Wollam, Dan Blackburn, George Devitt, Sarah Fearn, Christopher Kipps

**Affiliations:** 1 University of Southampton Faculty of Medicine, Southampton, UK; 2 National Institute for Health Research Collaboration for Leadership in Applied Health Research and Care Wessex, Hampshire, UK; 3 South Central Ambulance Service NHS Foundation Trust, Bicester, UK; 4 University of Warwick, Warwick Clinical Trials Unit, Coventry, UK; 5 Patient and Public Involvement and Engagement, NIHR Collaboration for Leadership in Applied Health Research and Care Wessex, Southampton, UK; 6 The University of Sheffield Institute for Translational Neuroscience, Sheffield, UK; 7 Neurodegenerative Research, University Hospital Southampton NHS Foundation Trust, Southampton, UK; 8 University Hospital Southampton NHS Foundation Trust Wessex Neurological Centre, Southampton, UK

**Keywords:** Dementia, NEUROLOGY, Community-Based Participatory Research, Feasibility Studies

## Abstract

**Abstract:**

**Introduction:**

Despite national efforts to improve research inclusion, people from underserved communities remain underrepresented in dementia trials. Barriers occur at the point of initial engagement and also within the participation pathway itself, as the structure and burden of early screening procedures can discourage continuation. ACCESS D (Advancing Community Collaboration and Engagement Strategies in Dementia) aims to address these challenges by testing a community-based model that combines co-produced engagement events, low-burden research participation, and real-time support from the South Central Ambulance Service (SCAS), a trusted, community-visible National Health Service (NHS) healthcare workforce serving the counties of Hampshire, Oxfordshire, Buckinghamshire and Berkshire in Southern England, UK.

**Methods and analysis:**

ACCESS D is a 12-month mixed-methods feasibility study recruiting 100 adults aged 50–90 years with either (1) a diagnosis of mild cognitive impairment or dementia or (2) a self- or proxy-reported memory concern affecting daily life. The study will deliver between 12–18 co-produced community outreach events in non-clinical settings, supported by SCAS research paramedics and nurses. Following written (paper or digital) informed consent, participants will complete a core questionnaire and may optionally take part in one or more low-burden research opportunities designed to provide supported, first-hand experience of dementia research. Feasibility outcomes, including pathway progression and opt-in to future dementia research contact, will be descriptively summarised and stratified using National Institute of Health and Care Research (NIHR) INCLUDE-aligned underserved characteristics. Qualitative interviews and focus groups with participants and staff will examine acceptability, perceived value, barriers and enablers and implementation learning, analysed using thematic analysis and integrated with quantitative findings.

**Ethics and dissemination:**

The study has received a favourable opinion from the Southwest-Frenchay Research Ethics Committee and Health Research Authority approval (IRAS 361074). Findings will be disseminated via peer-reviewed publications, conference presentations and co-produced lay outputs for community partners and participants. These outputs will be accompanied by an implementation toolkit for research teams and a visual summary for potential participants.

**Strengths and limitations of this study**

- This study tests a community-based, co-produced delivery model that embeds real research participation within outreach, addressing both awareness and structural barriers to dementia research participation.
- Outreach is delivered by a trusted, community-visible healthcare workforce, enabling real-time support and reducing psychological, practical and digital barriers for underserved groups.
- The mixed methods feasibility design combines quantitative indicators of reach, progression and resource use with qualitative insights into participant and staff experience, generating actionable learning for scale-up.
- Outcomes are explicitly equity-stratified using National Institute of Health and Care Research (NIHR) INCLUDE aligned characteristics, allowing early assessment of differential reach and engagement across underserved populations.
- As a single-region feasibility study, findings may have limited immediate generalisability; however, the study is designed to generate transferable implementation insights and inform a future multisite evaluation.

## Background and rationale

 Dementia affects over 980 000 people in the UK and 57 million worldwide, with numbers expected to double by 2050.^1^ It disproportionately affects older adults, but its impact is felt much more widely, among families, caregivers, communities and healthcare systems. The economic cost is immense; it costs the UK over £42 billion annually^2^ and global costs are predicted to rise from US$1.3 trillion to US$2.8 trillion by 2030.^3^

Research and clinical trials are essential to advance diagnosis, care and treatment, yet participation remains low and non-diverse. Barriers include mistrust, low awareness, stigma, fear of diagnosis and limited access to research opportunities, particularly in underserved populations where dementia risk is often higher.^4^ Traditional research models, largely based in hospitals or academic centres, increasingly focus on early-stage disease, when symptoms are subtle or mistaken for normal ageing. And even when outreach raises awareness, many people disengage due to the complexity, timing or intrusiveness of early screening.^5^,^8^

As a result, dementia research participants tend to be predominantly White, highly educated and already positive about research.^5 9^ This creates a cycle of exclusion. Communities most affected by dementia miss out; not only on the chance to influence future care and access new treatments but also on the personal benefits of participation.^10 11^

These inclusion gaps reduce the efficiency and generalisability of dementia research.^12 13^ For people affected by dementia, this contributes to slower innovation, inequitable access to advances and interventions that may not perform optimally across diverse populations, undermining the effectiveness of dementia care and the overall resilience of healthcare systems. Moreover, as dementia research increasingly prioritises early-stage and prevention trials, understanding how people enter, experience and progress through research participation pathways has become critical, aligning with key priorities of the National Institute of Health and Care Research (NIHR) INCLUDE Framework, the Dementia Mission and the UK Life Sciences Vision.

Advancing Community Collaboration and Engagement Strategies in Dementia (ACCESS D) addresses these longstanding structural and equity barriers by developing and testing a participant-centred, community-based model. Rather than expecting people to navigate to research opportunities within clinical and academic settings, ACCESS D brings dementia research directly to diverse community environments, making research more accessible and less intimidating.

Previous studies have shown that community-led strategies can increase participation by building trust, using culturally relevant messaging and involving local champions.^12^,^17^ However, persistent gaps remain, especially among people with lower income, lower educational attainment or limited understanding of research.^18^,^20^ Misunderstandings between research and clinical care and uncertainty about the benefits of participation remain common.^21^,^23^ As dementia research increasingly explores risk disclosure and prevention, transparent and accessible communication is essential to support informed decision-making.^24 25^

ACCESS D responds to these challenges by co-developing inclusive community events with people affected by dementia, public contributors (Patient and Public Involvement, Engagement and Participation, PPIEP), academic researchers and the South Central Ambulance Service (SCAS) research team. Events are delivered in familiar locations, including areas of increased socioeconomic deprivation and communities historically underrepresented in dementia research.

A persistent structural gap exists within research pathways between raising awareness of research and enabling meaningful participation. ACCESS D seeks to bridge this gap by embedding opportunities for research participation within community engagement events, supported in real time by the SCAS research team. This approach allows participants to experience what dementia research involves in a supported, low-pressure environment, helping to reduce both psychological and structural barriers to participation and create more equitable entry points into dementia research pathways.

High screen failure and early drop-out are well recognised in dementia research, particularly in early-phase and prevention trials. These inefficiencies can be frustrating for participants who have invested time and hope and are costly for research teams.^26^ By offering low-burden, supported research early in the engagement pathway, ACCESS D examines where interest develops or stalls and whether real-time support can help align expectations, improve acceptability and sustain participation. The study therefore aims to generate feasibility data to inform strategies for reducing screen failure and early dropout while supporting pathway progression.

ACCESS D is designed to generate learning in both directions. For community members, it offers a safe, non-clinical space to ask questions, experience research first-hand and share views on dementia, perceived risk and study design. For the research system, it generates structured insight into who is reached, what support is needed, which elements are acceptable and where barriers persist across the participation pathway. In parallel, ACCESS D will generate feasibility and process data on resource use and deliverability of the model. These data are intended to inform future programme-level funding, guide decisions about wider implementation and support adaptation of the model to other conditions and settings.

Dementia is used as a test case to evaluate the broader ACCESS framework: a flexible, community-first approach designed to improve equity and efficiency in research participation across conditions. The framework is underpinned by three interdependent principles:

1. Co-production with communities to ensure cultural relevance and legitimacy.
2. Delivery by trusted messengers to enhance credibility and approachability.
3. Low-burden, supported research participation to reduce barriers and build confidence.

By explicitly testing these principles in practice, ACCESS D aims to contribute transferable evidence to support decentralised, inclusive research delivery aligned with national priorities.

## Study aim and objectives

### Aim

To evaluate whether community-based outreach and support can make access to dementia research opportunities more inclusive and more efficient.

### Objectives

- Co-develop and pilot ways of engaging underserved communities that reduce barriers and help people consider taking part in dementia research.
- Explore the feasibility and acceptability of low-burden research participation offered within community events, such as questionnaires, interviews, digital tests and finger-prick samples, and their impact on confidence and research readiness.
- Assess early indicators of reach, pathway progression and scalability to inform future strategies for more inclusive and efficient recruitment.

## Methods and analysis

### Study design

ACCESS D is a 12-month mixed-methods feasibility study designed to test the feasibility, acceptability and implementation of a community-based model which aims to improve the equity and efficiency of dementia research participation.

In ACCESS D, ‘efficiency’ refers to early pathway progression and resource use. It will be assessed using stage-specific conversion rates (exposure/interest → consent → completion of ≥1 activity → opt-in to future contact) and delivery resource metrics (staff time and direct delivery costs per consented participant and per opt-in).

**Figure 1:**
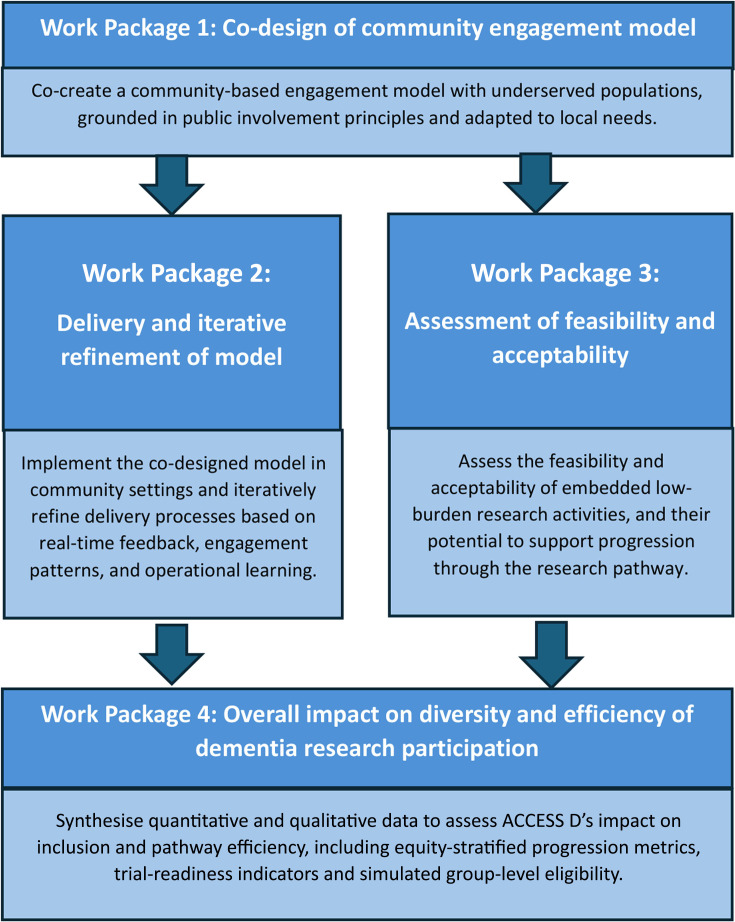
Conceptual overview of the ACCESS D Work Package structure. ACCESS D, Advancing Community Collaboration and Engagement Strategies in Dementia.

The study is structured around four linked Work Packages (WP; see figure 1):

Ethics, data-sharing, contracting and study set-up took place between September 2025 and February 2026.

#### WP1: co-produce a community-based engagement model

This WP (February to April 2026) involves co-developing an outreach model with people from underserved communities, public contributors, the SCAS research team and academic researchers. A minimum of three co-production workshops will be held in accessible, non-clinical community settings. These sessions will explore culturally relevant messaging, inclusive event design, trust-building strategies and barriers to research participation. Live illustration will be used to support accessibility. Outputs will include a co-produced event format, a visual summary for participants and a delivery toolkit for delivery teams. The toolkit will synthesise learning from co-production activities and is intended for research teams, community partners and research delivery staff. It will include practical guidance on community engagement approaches, accessible communication, delivery considerations, participant support, trust-building strategies, outreach planning and approaches to improving inclusivity and pathway progression within dementia research participation. Co-production workshops will be conducted in parallel with early outreach, allowing iterative refinements to events based on co-production feedback.

#### WP2: implement and refine the outreach model

Between 12–18 dementia-focused community outreach events will be delivered over 6 months (from February to July 2026) across diverse locations (eg, faith centres, public events, community groups, older people’s residential complexes and care homes). Events will be led by the SCAS research team and offer attendees opportunities to learn about dementia, ask questions and optionally take part in research (eg, questionnaires, testing a new digital cognitive assessment (CognoMemory), finger-prick sampling for biomarkers). Real-time feedback will be captured via debriefs and implementation logs to support ongoing refinement of the model.

#### WP3: evaluate feasibility, acceptability and value

This WP will occur over 6 months (from February to July 2026) in parallel with WP2, and will assess the feasibility, acceptability and perceived value of offering embedded and supported research opportunities in community settings. Data sources include postevent questionnaires (Likert and free-text), interviews and focus groups with participants, and reflective interviews with SCAS staff. Findings will explore motivators, barriers and how supported research activities shape understanding and confidence around research participation and sustained engagement.

#### WP4: evaluate progression and diversity across the participation pathway

The final WP (June to September 2026) will synthesise data to assess the model’s impact on both diversity and efficiency. This includes analysis of pathway progression (eg, consent and completion rates), dropout points and simulated trial eligibility. Outcomes will inform the design of more inclusive dementia research pipelines and provide a foundation for future scale-up.

At the time of manuscript submission in May 2026, ethics and HRA approvals had been obtained, data-sharing and delivery arrangements were in place, co-production activities had commenced and 10 outreach events had been delivered. Participant interviews, focus groups and data analysis in WP4 had not yet commenced.

### Setting

ACCESS D will be delivered across non-clinical community venues in Southampton and surrounding areas. Southampton had an estimated resident population of 259 424 in 2024, with 13.7% aged 65 years and over, lower than the England average of 18.7%. The city is relatively deprived, ranking 76th most deprived of 296 English local authorities in 2025, and is ethnically and linguistically diverse: 31.9% of residents identify as other than White British, 15.4% do not have English as their main language and nearly 160 languages are spoken locally.^27^

Census 2021 data indicate a mixed educational profile in Southampton. Among usual residents aged 16 years and over, 17.3% had no formal qualifications, 9.5% had level 1 or entry-level qualifications, 12.6% had level 2 qualifications, 5.4% had an apprenticeship as their highest qualification, 20.5% had level 3 qualifications and 31.6% had level 4 or above qualifications. In Census 2021, level 1 broadly corresponds to one to four General Certificate of Secondary Education (GCSE) passes or equivalent, level 2 to five or more GCSE passes or equivalent, level 3 to two or more A levels or equivalent and level 4 or above to higher education qualifications such as Higher National Certificate/Higher National Diploma, degree, postgraduate or professional qualifications. These figures are broadly similar to the national profile for England, although Southampton has a slightly lower proportion with level 4 or above qualifications than England overall and a higher proportion with level 3 qualifications.^28^

Local dementia need is substantial: in 2025, 1917 people aged 65 years and over had a recorded dementia diagnosis, while the estimated number living with dementia was higher at 2480, projected to rise to 3581 by 2045.^29^ Although local digital literacy estimates are limited, digital inclusion is identified as a local strategic priority, and national data suggest that 15% of UK adults lack foundation-level essential digital skills and 3% are offline.^30 31^

Venues will be selected co-productively with public contributors, community partners, research paramedics and nurses and the academic team to maximise accessibility, cultural relevance and inclusion. Likely settings include community centres, places of worship, libraries, public events and other community-facing locations, including care homes and older people’s residential complexes so as to provide accessibility for those with lower mobility and limited access to transport. Venue suitability (including privacy and the feasibility of on-site research activities) will be assessed in advance through risk assessment led by SCAS. Where required, a SCAS research ambulance will provide a mobile private space for consenting and optional finger-prick sampling.

### Patient and public involvement and engagement

ACCESS D has been informed by previous public engagement and community dialogue work. Deliberative dialogue workshops conducted as part of a previous research project involved a purposive sample of 50 members of the public from diverse communities and highlighted limited public understanding of what research involves and what researchers actually do. Community outreach conducted by the SCAS research team across a range of local groups and communities, including engagement with the local Vedic Hindu temple, revealed strong support for bringing research into familiar community settings and providing opportunities for face-to-face discussion in a supported, low-pressure environment.

Two public contributors reviewed and informed participant-facing documentation. One contributor was of mixed Asian ethnicity with personal experience of dementia and extensive experience of community engagement with local ethnic minority communities. The second contributor was of White British ethnicity with personal and professional experience of dementia. Their input informed the use of plain English materials, summary and detailed participant information sheets, accessibility considerations and community-based delivery.

Public contributors will continue to contribute throughout the study through involvement in interpretation of findings, refinement of outreach approaches and dissemination activities, including co-produced lay summaries, presentations and peer-reviewed publications.

### Recruitment pathways

**Figure 2:**
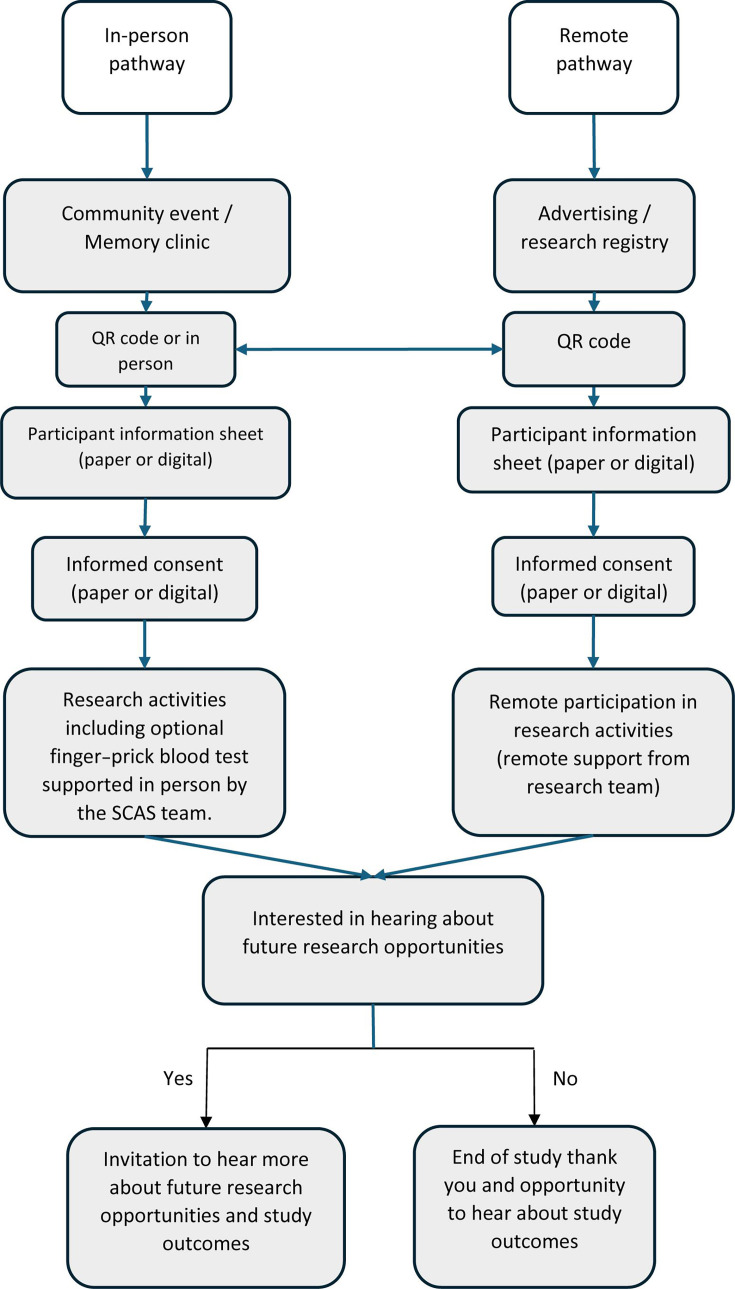
ACCESS D participation pathways. ACCESS D, Advancing Community Collaboration and Engagement Strategies in Dementia; SCAS, South Central Ambulance Service.

To maximise reach and enable descriptive comparison of engagement profiles, ACCESS D will recruit participants via four prespecified pathways (see figure 2). These pathways are specified to allow descriptive comparison of reach, acceptability and progression across different entry points into research.

1. Community events: in-person recruitment in community venues led by the SCAS research team, with optional on-site research participation.
2. National Health Service (NHS) memory clinics: clinical teams introduce the study to potentially eligible patients, including those who are hesitant to enrol on a trial but would like to learn more about research before committing; individuals can enrol online and/or attend a community event.
3. Research registries: recruitment via existing platforms (eg, Join Dementia Research).
4. Online recruitment: adverts and leaflets linking to online study information and consent.

Participants may move between pathways. For example, someone who registers online may choose to attend a community event, or a memory clinic patient may opt to participate remotely. These transitions will be recorded and disaggregated in analysis. The target of 100 participants is cumulative across all routes.

### Outreach events and implementation

Between 12–18 outreach events will be delivered over approximately 6 months. Events will vary in duration (approximately 2–6 hours) depending on venue type and footfall (eg, organised group sessions vs public pop-ups). At events, attendees can:

- learn about dementia and dementia research using NHS-approved materials,
- speak with the SCAS research team in an informal setting, and
- optionally consent and take part in study activities with real-time support.

Participation in research activities is voluntary; attendees may engage with events without taking part in any research. The delivery format will be iteratively refined using implementation logs, event feedback and debriefs to identify barriers, improve acceptability and optimise accessibility.

### Participants

#### Eligibility criteria

Participants will be enrolled into two broad groups:

- Group A: Those with a diagnosis of mild cognitive impairment (MCI) or dementia. This group includes individuals with established clinical diagnoses, to understand engagement in populations currently accessing clinical care
- Group B: Those with memory concerns but no formal diagnosis.

#### Inclusion criteria

- Aged 50–90 years (inclusive).
- Group A: clinical diagnosis of MCI or dementia.
- Group B: self-reported or proxy-reported memory concerns perceived to affect daily life.

#### Exclusion criteria

- Lack of capacity to provide informed consent at enrolment.
- Any medical, psychiatric or behavioural condition which, in the opinion of research staff, would make participation unsafe.

### Staff

Staff eligible for interviews or focus groups will include SCAS research paramedics, nurses and other delivery staff directly involved in planning, delivering or supporting ACCESS D outreach events or study procedures. Staff must be aged 18 years or over, able to provide informed consent and have sufficient experience of study delivery to contribute reflective feedback. Staff not directly involved in ACCESS D delivery will not be eligible.

### Capacity and informed consent

All participants must have capacity to provide informed consent, assessed in accordance with the Mental Capacity Act (2005). Capacity will be assumed unless there is evidence to the contrary. Trained research staff will ensure participants can understand, retain and weigh the study information and communicate a voluntary decision.

Written informed consent will be given before any study activities. Participants may consent in person (paper or electronic) at outreach events, or remotely via a secure online platform accessed through a QR code. Individuals can take information away and decide in their own time, and can ask questions at any point. Participants may withdraw at any time without giving a reason; where feasible, anonymised reasons for withdrawal will be recorded to inform feasibility and improvement.

No financial incentives will be offered. Refreshments may be provided at community events. Travel expenses will be paid to those participants opting for an in-person interview or focus group.

### Enhancing inclusivity

Inclusivity will be supported through parallel recruitment and participation pathways, including community events, memory clinic signposting, registry recruitment and remote participation.

Venues will be selected to maximise physical accessibility, including consideration of step-free access, accessible toilets, parking, public transport links and private space for consent or study activities. Participants who are unable to attend a community event may complete eligible study activities remotely, by telephone, by paper copy or with support from the research team where feasible. Events will also be held in care homes and residential complexes to increase inclusivity for those without transport or with mobility constraints.

Participant-facing materials have been reviewed by PPIEP contributors and written in plain English. Both summary (Gunning Fog <9) and detailed participant information sheets will be available, with paper and large-print versions provided on request. At outreach events, SCAS research staff will provide real-time support, including explaining study activities, helping participants navigate digital tools, supporting questionnaire completion and answering questions. Participants choosing to participate remotely will be able to contact the research team for support.

Reasonable adjustments will be made wherever feasible. For participants with visual impairment, support will include large-print materials, paper copies, verbal administration of questionnaires and researcher-supported completion. For participants with hearing impairment, interviews and discussions may be conducted face-to-face in quiet spaces, with written prompts or written responses, video calls with captions where feasible, or support from a family member or carer if requested by the participant. Quiet spaces will also be available for participants who require a lower-stimulation environment.

Events will be delivered in English, but NHS-approved dementia information leaflets in multiple languages will be available. For participants whose first language is not English, research staff will explain the study in simple terms and may involve family members or supporters where requested by the participant. The CognoMemory assessment is currently available only in English, and the validated Everyday Cognition Questionnaire (ECog) is available in English. Due to budget constraints, formal translation or interpreting services cannot be provided. Where language barriers mean that informed consent cannot be established, individuals will not be enrolled in the research component; however, they may still access general dementia information and signposting at the event. Unmet language needs that limit participation will be documented to inform future phases.

Digital inclusion will be supported through in-person participation routes, paper-based materials, staff-supported completion at events and remote support by telephone or email. Where feasible, audio versions of questionnaire materials will be created. Participants will not be excluded solely because of hearing, visual, mobility, language or digital access needs, provided informed consent can be obtained and study activities can be completed safely and meaningfully with reasonable adjustments.

Recruitment data will be monitored throughout the study to assess reach into under-represented groups. Engagement strategies will be iteratively adjusted in collaboration with PPIEP contributors and community partners to maximise inclusivity and representativeness.

### Study procedures and data collection

Study procedures and data collection are summarised in table 1.

**Table 1:** Summary of ACCESS D study activities, settings and timing

Activity	Setting	Timing	Data captured
Participant information sheet and informed consent	Community event Memory clinic Registry Fully remote	Enrolment	Paper or electronic consent
Core questionnaire	Paper, event tablet or personal device	Post consent	Primary outcome data, including demographics, ECog-12/experience/acceptability/views on memory, dementia risk and research design.
Digital memory test	Event device or personal device	Post consent	Summary outputs from CognoMemory
Finger-prick sample	Community events only	Post consent	Exploratory biomarkers including, for example, amyloid proteins
Focus group/interview	Remote or in-person	Post participation	Purposive sampling to explore acceptability, including underserved status and those who decline research activities
6-month follow-up	Remote using preferred mode of contact	Approx 6 months post participation	Self-report of subsequent research participation

ACCESS D, Advancing Community Collaboration and Engagement Strategies in Dementia; ECog-12, 12-Item Everyday Cognition Questionnaire.

### Overview of study activities

Following informed consent, participants will be assigned a unique study ID and offered:

- a core study questionnaire (all participants), and
- optional research participation opportunities (participants may choose any, all or none).

### Core activity: questionnaire

All participants will be offered a mixed-format questionnaire (Likert and free-text) available in paper or digital format, with staff support as needed. The questionnaire covers:

- experience and acceptability of the outreach event
- perceived barriers and enablers to dementia research participation
- willingness to take part in future research
- views on dementia risk and research design
- self-reported memory concerns/diagnoses
- representation variables (age, gender, ethnicity, education, digital confidence, postcode to derive Index of Multiple Deprivation (IMD))
- the ECog-12
- consent preferences for future research contact and optional follow-up.

The core questionnaire was developed specifically for ACCESS D to capture feasibility, acceptability, barriers and enablers to dementia research participation, willingness to take part in future research, demographic characteristics and future contact preferences. Items were informed by the study aims, NIHR INCLUDE-aligned equity characteristics, prior public engagement work, PPIEP review and relevant literature on research participation and acceptability. The questionnaire includes a combination of Likert-scale and free-text items and incorporates the ECog-12 as a validated measure of everyday cognition. The questionnaire has been reviewed by public contributors for clarity, acceptability and accessibility and is included as an online supplemental file. As this is a feasibility study, the ACCESS D-specific items are not intended as a validated scale; their performance, completeness and acceptability will be assessed to inform refinement for future studies.

### Optional research participation opportunities

Following completion of the core questionnaire, participants may choose to take part in one or more optional research activities. These activities are intended to provide supported, low-burden examples of procedures commonly used in dementia research and trials. They are not being evaluated for clinical validity within ACCESS D and will not be used for diagnosis, clinical decision-making or individual eligibility assessment.

Digital memory assessment (CognoMemory):

1. CognoMemory is a web-based, speech-driven memory assessment tool developed by the University of Sheffield currently undergoing validation. It uses a virtual agent to administer brief memory and language-based tasks, such as picture description, verbal fluency and recall, and applies automated speech and language analysis to generate cognitive summary outputs.^32 33^ The assessment takes approximately 10 min to complete. Participants interact with an on-screen virtual agent, either in-person at outreach events or remotely using a personal device. A separate digital consent form is embedded within the CognoMemory platform, as required by its independent ethical approval. Data from the assessment will be processed and managed by the University of Sheffield under its own ethical approval and governance arrangements. Pseudonymised summary scores will be shared with the Southampton research team following data sharing agreements and via secure transfer methods approved by both the University of Southampton and Sheffield. The scores will be used in a group-level exploratory simulation to determine how well ACCESS D can increase diversity in trials, in the absence of an actively recruiting trial during the study period.
2. Finger-prick blood sampling (events only): participants attending in person may opt to provide a finger-prick blood sample for dementia biomarkers, collected by the SCAS research team in an appropriate private setting. Finger-prick blood sampling is included primarily to assess the feasibility and acceptability of collecting these blood samples in community settings, rather than to evaluate biomarker performance or return individual results. Samples will be used for exploratory measurement of biomarkers relevant to dementia research, such as amyloid, tau and/or neurodegeneration-related markers, subject to sample volume and assay feasibility. Blood-based biomarkers are increasingly used in Alzheimer’s disease research and are being evaluated for clinical implementation,^34^ while finger-prick and dried blood spot approaches are emerging as minimally invasive collection methods.^35 36^ Our analyses will be conducted at group level only to explore feasibility, sample quality and to determine how well ACCESS D can increase diversity of trial participation, in the absence of an actively recruiting trial during the study period. Results will not be used for diagnosis, clinical decision-making or individual eligibility assessment, and individual biomarker results will not be returned to participants, as clearly detailed in participant information sheets.
3. Qualitative interview or focus group: a purposive subsample of participants will be invited to take part in an interview (up to 45 min) or focus group (up to 60 min), conducted in person, online or by telephone. Sessions will be audio-recorded with consent, transcribed and analysed thematically. Sampling will include participants from underserved groups and those who declined optional activities.
4. 6-month follow-up: participants may opt to receive a brief follow-up communication via their preferred route eg email, post, phone or text, approximately 6 months after participation asking whether they have taken part in other dementia research since ACCESS D.

### Role of illustrative research activities

Research activities offered within ACCESS D are not being formally evaluated for clinical validity. Instead, they serve two distinct purposes: (1) as low-burden examples of typical dementia research procedures, enabling participants to experience deconstructed trial components (eg, interviews, questionnaires, digital cognitive tests, finger-prick sampling) in a safe, supported setting and (2) to generate feasibility data on delivery, acceptability and uptake in community settings, and to explore whether the ACCESS D model can increase the diversity and efficiency of research participation.

These activities were selected because they map onto issues that matter to people affected by memory concerns. Long waits for memory assessment and diagnosis can create uncertainty and anxiety; the 2023/24 National Audit of Dementia reported an average wait of 151 days from referral to diagnosis, ranging from 44 to 347 days across services.^37^ CognoMemory illustrates research aiming to support more efficient assessment pathways, while finger-prick sampling illustrates how less invasive approaches may, in future, support diagnosis and monitoring. In ACCESS D, both activities are exploratory, non-diagnostic and individual results will not be returned, but aim to demonstrate how worthwhile research can be.

### Staff interviews and implementation data

SCAS research team staff involved in delivery will contribute implementation insights through debrief discussions, event evaluation logs and semistructured interviews. Topics will include feasibility of delivery, workload, barriers and enablers, participant support needs, and recommendations for optimisation and scale-up. A purposive sample of approximately five staff members will be invited to ensure a range of delivery perspectives and experiences.

### Outcomes

#### Primary outcome: progression to future research contact

The primary outcome is the proportion of consented participants who opt in to be contacted about future dementia research opportunities.

- Numerator: number of participants providing consent for future research contact.
- Denominator: all consented participants.

This outcome was selected as a key indicator of feasibility because it reflects a key transition from initial engagement to potential longer-term involvement in research without relying on the availability of a specific downstream trial. It captures participants’ willingness to remain connected to research and helps assess whether the ACCESS D model can support sustained research readiness among diverse and underserved populations.

#### Co-primary outcome: equity-stratified primary outcome

The primary outcome will be stratified by underserved status (NIHR INCLUDE–aligned criteria) and by recruitment pathway to assess whether ACCESS D reaches and engages groups historically underrepresented in dementia research.

For analysis, participants will be classified as ‘underserved’ if they meet ≥1 of the following prespecified criteria: (1) IMD deciles 1–3 derived from participant home postcode (socioeconomic deprivation); (2) minority ethnic background (UK standard ethnicity categories; self-report); (3) low digital confidence (self-report) and (4) low educational attainment (self-report). In addition, we will derive an underserved score (0–4) as the count of criteria met to support descriptive summaries of intersectionality.

#### Secondary outcomes

Secondary outcomes will be analysed descriptively and will be stratified by underserved group status where numbers permit.

#### Process efficiency

- Activity uptake: proportion of consented participants completing ≥1 study activity (questionnaire and an optional activity).
- Delivery fidelity: proportion of planned outreach events delivered.
- Event yield: number of participants consented per outreach event and number completing ≥1 activity per event.

#### Staff experience

- Staff acceptability: proportion reporting a positive postevent experience (≥7/10).
- Staff time: staff hours per event, by role.
- Qualitative staff experience: themes from staff interviews, debriefs and delivery logs.

#### Participant acceptability

- Overall experience: proportion rating experience as positive (≥7/10).
- Recommendation: proportion willing to recommend participation to others.
- Support needs: proportion requiring assistance to complete any activity.
- Qualitative participant experience: themes relating to trust, burden, clarity, perceived value and barriers.

#### Pathway efficiency (within-participant progression)

- Stage-specific conversion rates across the participation pathway (exposure/interest → consent → completion of ≥1 activity → opt-in to future contact → optional 6-month follow-up response), summarised overall and stratified by recruitment pathway.

#### Recruitment pathway yield (exploratory)

- Exploratory analysis of opt-in rates by recruitment pathway to provide indicative signals of yield. Denominators are defined as:
  - Community events: number of individuals who engage with the outreach team and accept study information (leaflet/QR card) as recorded in event logs.
  - NHS memory clinics: number of study leaflets distributed (clinic log).
  - Research registries (eg, JDR): number of invitations issued (and, where available, number opened/clicked) as provided by the registry platform.
  - Online recruitment: number of unique study-page visits (and, where available, impressions and click-throughs) from advertisement analytics.

#### Trial eligibility simulation (exploratory)

- Group-level simulation of eligibility for exemplar dementia trials using questionnaire data, demographics, and optional activity data (further described in Data Analysis).

#### Longer-term engagement (exploratory)

- Response to optional 6-month follow-up and self-reported participation in other dementia research since ACCESS D.

#### Resource use and scalability (exploratory; at event and project level)

- Staff hours (by role)
- Direct delivery cost
- Cost per consented participant
- Cost per opt-in to future research contact

### Sample size

This is an exploratory feasibility study; therefore, no formal sample size calculation has been conducted. A pragmatic target of 100 participants typical of feasibility studies has been set to assess feasibility and acceptability in diverse community settings and to provide initial descriptive estimates of engagement and progression, including stratified summaries where subgroup sizes permit. For qualitative components, up to 20 participants and approximately 5 SCAS staff will be purposively sampled to ensure diversity and analytic adequacy. Across the WPs, WP1 will include at least three co-production workshops with approximately 8–12 contributors per workshop. WP2 will include up to 100 participants completing the core questionnaire and optional research activities. WP3 will also include up to 20 participant interviews/focus groups and approximately five staff interviews. WP4 will synthesise all available quantitative, qualitative and implementation data.

### Data analysis

#### Quantitative analysis

Quantitative analysis will be descriptive, with no hypothesis testing. Analyses will be conducted using STATA 18 (or equivalent). Categorical variables will be summarised using frequencies and proportions; continuous variables using means and SD or medians and IQRs, as appropriate. Where subgroup numbers permit, outcomes will be stratified by underserved group status and recruitment pathway. Missing data will not be imputed; patterns of missingness, non-completion, withdrawal and loss to follow-up will be described to inform the design, delivery and retention strategies of future studies.

#### Exploratory group-level simulation of trial eligibility

In the absence of an actively recruiting dementia trial during the study period, data collected within ACCESS D (eg, age, ECog-12 and optional measures such as CognoMemory summaries and biomarkers) will be used in an exploratory, group-level simulation. This simulation will estimate the proportion of participants who would theoretically meet pre-specified exemplar dementia trial eligibility criteria, selected to reflect common inclusion and exclusion features of early-phase dementia trials, and to characterise patterns of potential exclusion.

This analysis is intended solely to inform understanding of eligibility constraints at a population level. It will not be used to make individual eligibility determinations, and no individual results will be returned to participants.

### Qualitative analysis

Free-text survey responses and interview/focus group transcripts of participants and staff will be analysed using inductive thematic analysis following Braun and Clarke’s six-phase approach.^38^ NVivo and/or Excel will support data management. Interpretation will be guided by relevant theoretical lenses, used to contextualise findings rather than as prescriptive coding frameworks, including Normalisation Process Theory and the Theoretical Framework of Acceptability, to explain acceptability and implementation across contexts (ie, what works, for whom and in what settings).

Interviews and focus groups will be conducted in person, online or by telephone according to participant preference and feasibility. Interviews, focus groups and qualitative coding will be conducted by PF, a medically trained mixed-methods member of the research team with a Master’s degree in Public Health and 3 years’ experience in qualitative and mixed-methods research. A subset of transcripts and coding will be reviewed by SF, a qualitative researcher in the research team with 17 years’ experience, to support rigour. Rapport will be supported through plain-language explanations, flexible modes of participation, time for questions, and sensitivity to participant preferences and support needs. At the start of interviews and focus groups, it will be emphasised that both positive and negative feedback are welcome and that there are no right or wrong answers. Reflexive notes will be maintained throughout data collection and analysis to support transparency regarding researcher perspectives and interpretation of findings. Qualitative sampling will be purposive and iterative. As this is a feasibility study, recruitment will be guided by diversity of perspectives and data adequacy rather than formal theoretical saturation.^39 40^ The research team will monitor whether interviews and focus groups generate sufficient depth and breadth to address the study aims, including perspectives from underserved groups and those declining optional activities.

### Mixed-methods integration

A convergent mixed-methods design will be used, whereby quantitative and qualitative data are collected during the same feasibility phase, analysed separately and then integrated during interpretation.^41^ Integration will use triangulation to compare areas of agreement, complementarity and divergence across quantitative findings, qualitative findings and implementation data, including event debriefs, delivery logs, field notes and staff reflections. A triangulation matrix will support systematic comparison across data sources to identify how different forms of evidence contribute to understanding feasibility, acceptability, equity, implementation challenges and optimisation of the ACCESS D model across different settings and participant groups.

### Study management, governance and ethics

ACCESS D will be overseen by a Study Management Group (SMG) comprising the Chief Investigator, the study coordinator and lead applicant, SCAS research paramedic delivery lead and PPIEP contributors. The SMG will oversee study delivery, recruitment and equity monitoring, participant safety, data quality and documented iterative refinements to the delivery model.

The study has received a favourable opinion and approval from the Southwest-Frenchay Research Ethics Committee and the Health Research Authority. Any protocol amendments will be submitted for REC/HRA approval as required prior to implementation.

ACCESS D will comply with the UK General Data Protection Regulation, the Data Protection Act 2018, and sponsor and university information governance policies. All participants will be assigned a unique study identifier. Research datasets will be pseudonymised, with identifiable information stored separately and accessed only by authorised study personnel.

Consent and questionnaire data will be captured using secure, institutionally approved systems (eg, REDCap or Qualtrics). Access will be role-restricted, and analysis datasets will contain no direct identifiers. Data generated through the CognoMemory platform will be governed under separate ethical approval and consent processes; only minimised, pseudonymised summary outputs required for prespecified exploratory analyses will be shared with the ACCESS D team under appropriate data sharing agreements.

Participants opting into finger-prick sampling will provide samples labelled with study ID only, processed in accordance with sponsor and laboratory governance requirements. Qualitative interviews and focus groups will be audio-recorded with consent, transcribed, de-identified and stored securely.

ACCESS D is designed to minimise risk. Optional study activities are research-only and non-diagnostic, and no individual results will be returned to participants, as detailed in the participant information sheets. Staff will follow a distress protocol to identify and respond to emotional discomfort, including pausing activities, reaffirming voluntariness and signposting to appropriate support. Participants may withdraw at any time without consequence; unless otherwise requested, data collected up to withdrawal will be retained in pseudonymised form.

Findings will be disseminated through peer-reviewed publications, conference presentations, reports to funders and local partners, and co-produced lay summaries for participants and community organisations. Outputs will also include a visual participant summary and an implementation toolkit for research teams, NHS organisations, community partners and research delivery staff.

## Discussion

ACCESS D is a mixed-methods feasibility study designed to address persistent inequities and inefficiencies in dementia research participation. It combines co-produced community outreach, delivery by a trusted healthcare workforce, and real-time, supported opportunities to experience low-burden research activities typical of dementia studies and trials. The goal is to make research more accessible, better understood and more inclusive, particularly for people from underserved communities and those not yet known to memory services.

The study will generate detailed feasibility and process data on reach, pathway progression, equity and resource use, alongside qualitative insights into participant and staff experience to ensure acceptability. Together, these data will provide an integrated understanding of how community-based delivery models function in practice, including which elements are acceptable, where support is most needed and how participation pathways differ across populations and recruitment routes. This evidence is intended to inform future programme-level applications and to provide practical guidance for embedding community-based, equitable and efficient approaches within dementia research infrastructure.

This feasibility study has several limitations. It is conducted in one region of southern England, which may limit immediate generalisability to other regions or differently configured research delivery systems. The sample size is pragmatic and not powered to test effectiveness or subgroup differences. Participants who attend community events or choose to enrol may be more interested in dementia research than those who do not engage, creating potential selection bias. Formal translation and interpreting services are not available within the study budget, which may result in under-representation of some non-English-speaking groups. All instances where language barriers prevent informed consent or limit participation will be systematically documented to characterise the extent of this limitation and inform the design and resourcing of future phases.

Following this feasibility phase, the next step is a multisite implementation study to test transferability across regions, delivery contexts and conditions. Future work will also explore data linkage to routine health records and research registries to assess sustained engagement and longer-term outcomes.

Although dementia is used as the primary test case, the core principles of community co-production, delivery by trusted messengers and low-burden, supported research participation are intentionally condition-agnostic. These principles are likely to be transferable to other long-term conditions, such as respiratory disease, where similar participation challenges exist. In this way, the model aligns with national priorities set out in the NIHR INCLUDE Framework, the Dementia Mission and the UK Life Sciences Vision by generating evidence to support more inclusive recruitment, minimise early attrition and screen failure, and enable more efficient research delivery.

## Supplementary material

10.1136/bmjopen-2026-118119 online supplemental file 1
