# Supplementary material for: Mixed-methods feasibility study of a community-based model to improve equity and efficiency in dementia research participation: protocol for ACCESS D
Source: BMJ Open. 2026 Jul 13;16(7):e118119. doi: 10.1136/bmjopen-2026-118119 (PMC13365783; doi:10.1136/bmjopen-2026-118119)
Supplement: online supplemental file 1 [file bmjopen-16-7-s001.docx]

**ACCESS D: *Advancing Community Collaboration and Engagement Strategies in Dementia***

**Questionnaire: Making dementia research more inclusive and appealing to everyone**

Version 1.0; 14/07/25; IRAS number 361074

**Thank you so much for being part of ACCESS D.**

- This short survey asks what you thought about taking part and what matters to you when it comes to dementia research.
- There are no right or wrong answers.
- There are five sections, and it will take about **15–20 minutes** for you to do. Please skip any question you're not comfortable answering.
- You can tell us if you’d like to receive a summary of what other people told us and what we’ve learned.

Thank you.

**Section 1: Your experience with ACCESS D**

Q1. Where did you hear about ACCESS D? (You can tick more than one option).

| ACCESS D community event |  |
| --- | --- |
| Ambulance staff |  |
| Online or social media |  |
| Memory clinic |  |
| GP |  |
| Family or friends |  |
| Join Dementia Research |  |
| Community group |  |
| Prefer not to say |  |
| Other: Please tell us here. |  |

Q2. Had you taken part in research before?

| Yes, several times | Yes, once or twice | I was invited, but didn’t take part | No, I had never heard about research before | Prefer not to say | Don’t know |
| --- | --- | --- | --- | --- | --- |
|  |  |  |  |  |  |

Q3. Why did you decide to take part in ACCESS D?   Tick up to three.

| I’m worried about my memory |  |
| --- | --- |
| The study was easy to take part in |  |
| I went to an ACCESS D event |  |
| I liked being able to try out different types of research |  |
| I trusted the ambulance staff |  |
| I wanted to help improve dementia research |  |
| I could choose between online or in-person |  |
| I liked that it could help my community |  |
| The team offered support and equipment to help take part |  |
| The research activities looked useful and meaningful |  |
| Prefer not to say / Don’t know |  |
| Other reason: Please tell us why here? | |

Q4. Which ACCESS D activities did you do? (Tick all that apply)

| Questionnaire | Interview | Digital Memory Test | Fingerprick blood test | Community event |
| --- | --- | --- | --- | --- |
|  |  |  |  |  |

Q5. Would you be happy to do any of the research options again?

|  | Yes | No | I didn’t do this | Prefer not to say |
| --- | --- | --- | --- | --- |
| Questionnaire |  |  |  |  |
| Interview |  |  |  |  |
| Digital Memory Test |  |  |  |  |
| Fingerprick blood test |  |  |  |  |

Q6. How did you complete the activities (excluding blood tests)?

| By myself | With help from a family member, friend or carer | With help from the research team | Prefer not to say |
| --- | --- | --- | --- |
|  |  |  |  |

Q7. I would recommend ACCESS D to others in my family, friends or community.

| Strongly agree | Agree | Neutral | Disagree | Strongly disagree | Don’t know | Prefer not to say |
| --- | --- | --- | --- | --- | --- | --- |
|  |  |  |  |  |  |  |

Q8. Overall, how would you rate your experience with ACCESS D? Please circle or tick a number.

| 0 | 1 | 2 | 3 | 4 | 5 | 6 | 7 | 8 | 9 | 10 |
| --- | --- | --- | --- | --- | --- | --- | --- | --- | --- | --- |
| Very bad |  |  |  |  | Neutral |  |  |  |  | Excellent |

Q9: Overall, how acceptable did you find taking part in ACCESS D?

| 0 | 1 | 2 | 3 | 4 | 5 | 6 | 7 | 8 | 9 | 10 |
| --- | --- | --- | --- | --- | --- | --- | --- | --- | --- | --- |
| Not at all acceptable |  |  |  |  | Neutral |  |  |  |  | Extremely acceptable |

Q10. Which parts of ACCESS D made the biggest difference to you deciding to take part and why? This could be things like the people you met, where it took place, how easy it was, or something else.

|  |
| --- |

Q11: Would you like a short summary of what this study finds?

| Yes | No | Don’t know | Prefer not to say |
| --- | --- | --- | --- |
| Please enter your contact here:  Text:  Phone:  Email: |  |  |  |

Q12: Would you like us to contact you about other dementia research you might like to take part in? You can ask us to delete your details at any time.

| Yes | No | Don’t know | Prefer not to say |
| --- | --- | --- | --- |
| Please enter your preferred contact method here:  Text:  Phone:  Email: |  |  |  |

**Section 2: What You Think About Dementia research and Brain Health**

Q1. Before ACCESS D, I already knew about dementia research.

| Strongly agree | Agree | Neutral | Disagree | Strongly disagree | Don’t know | Prefer not to say |
| --- | --- | --- | --- | --- | --- | --- |
|  |  |  |  |  |  |  |

Q2. Since taking part in ACCESS D, what changed for you? (Please tick all that apply)

| I understand dementia research better |  |
| --- | --- |
| I feel more confident talking about dementia |  |
| I learned something new about memory and brain health |  |
| I plan to make lifestyle changes to support my brain health |  |
| I will talk to family and friends about memory issues |  |
| I’d like to help others take part in research |  |
| I will consider joining future dementia studies |  |
| I will encourage others to take part in dementia research |  |
| I don’t plan to make any changes as a result of taking part |  |
| I see people like me in research materials |  |
| Research feels welcoming to me |  |
| I will not do any more research |  |
| None of these |  |
| Prefer not to say |  |
| Other change you’ve noticed: | |

Q3. How do you feel about your memory and thinking at the moment? Please tick all that apply.

| I have concerns about my memory or thinking skills |  |
| --- | --- |
| People close to me have noticed changes in my memory or thinking skills |  |
| I think my memory or thinking skills have got worse |  |
| I have been diagnosed with mild cognitive impairment |  |
| I have been diagnosed with Alzheimer’s disease |  |
| I have been diagnosed with another form of dementia |  |
| I take medication to help with memory and thinking problems |  |
| I have a family history of dementia (parent, sibling) |  |
| I have taken a memory test before |  |
| I am happy with my memory / thinking |  |
| None of these apply |  |
| Prefer not to say |  |
| I don’t know |  |

Q4. Some things can feel harder with time. The following questions ask about how your thinking skills have changed compared to roughly 10 years ago. Please answer as best as you can.

1. Finding things you have put down, for example keys or glasses

| No change | Occasionally worse | Consistently a little worse | Consistently much worse |
| --- | --- | --- | --- |
|  |  |  |  |

2. Remembering the date or day of the week.

| No change | Occasionally worse | Consistently a little worse | Consistently much worse |
| --- | --- | --- | --- |
|  |  |  |  |

3. Saying what you want to say in a conversation

| No change | Occasionally worse | Consistently a little worse | Consistently much worse |
| --- | --- | --- | --- |
|  |  |  |  |

4. Understanding spoken directions or instructions.

| No change | Occasionally worse | Consistently a little worse | Consistently much worse |
| --- | --- | --- | --- |
|  |  |  |  |

5. Needing navigational aids eg GPS or your phone to find your way around

| No change | Occasionally worse | Consistently a little worse | Consistently much worse |
| --- | --- | --- | --- |
|  |  |  |  |

| 6. Finding your way around places you know well (like your home) |
| --- |

| No change | Occasionally worse | Consistently a little worse | Consistently much worse |
| --- | --- | --- | --- |
|  |  |  |  |

7. Anticipating the weather, like taking a coat or umbrella

| No change | Occasionally worse | Consistently a little worse | Consistently much worse |
| --- | --- | --- | --- |
|  |  |  |  |

8. Planning a trip, holiday or day out

| No change | Occasionally worse | Consistently a little worse | Consistently much worse |
| --- | --- | --- | --- |
|  |  |  |  |

9. Keeping your home or workplace organised

| No change | Occasionally worse | Consistently a little worse | Consistently much worse |
| --- | --- | --- | --- |
|  |  |  |  |

10. Paying bills or managing payments.

| No change | Occasionally worse | Consistently a little worse | Consistently much worse |
| --- | --- | --- | --- |
|  |  |  |  |

11. Doing two things at once

| No change | Occasionally worse | Consistently a little worse | Consistently much worse |
| --- | --- | --- | --- |
|  |  |  |  |

12. Doing a task while talking to someone

| No change | Occasionally worse | Consistently a little worse | Consistently much worse |
| --- | --- | --- | --- |
|  |  |  |  |

Q5. I think simple lifestyle changes (like eating better, exercising) can lower my chances of getting dementia? Please tick.

| Strongly agree | Agree | Neutral | Disagree | Strongly disagree | Don’t know | Prefer not to say |
| --- | --- | --- | --- | --- | --- | --- |
|  |  |  |  |  |  |  |

Q6. What might stop you wanting to know your risk of dementia? Please tick all that apply.

| Anxiety or stress |  |
| --- | --- |
| I would rather focus on living well now |  |
| I wouldn’t know what to do next |  |
| I wouldn’t trust the result |  |
| I would rather wait until I had memory problems |  |
| I’d feel labelled or judged |  |
| I wouldn’t want to upset my family |  |
| Nothing would stop me |  |
| I don’t know |  |
| Prefer not to say |  |
| Other: please tell us here | |

Q7. How likely do you think you are to get dementia in the future? Please tick.

| Very likely | Somewhat likely | Neither likely nor unlikely | Not very likely | Not likely at all | Don’t know | Prefer not to say |
| --- | --- | --- | --- | --- | --- | --- |
|  |  |  |  |  |  |  |

**Section 3: Brain Health Tests and Research Preferences**

Q1. I’d be happy to have regular brain health check-ups by a healthcare professional. Please tick a box.

| Strongly agree | Agree | Neutral | Disagree | Strongly disagree | Don’t know | Prefer not to say |
| --- | --- | --- | --- | --- | --- | --- |
|  |  |  |  |  |  |  |

Q2: Would you be happy to take part in research that involves the following activities?

| **Type of research study** | **Yes** | **No** | **Not sure** | **Prefer not to say** |
| --- | --- | --- | --- | --- |
| A short questionnaire |  |  |  |  |
| A one-to-one or group interview |  |  |  |  |
| A diet or lifestyle programme |  |  |  |  |
| A study that has regular health checks on you |  |  |  |  |
| Wearing an activity or sleep tracker for a couple of weeks |  |  |  |  |
| Giving a blood sample |  |  |  |  |
| A spinal fluid test (lumbar puncture) |  |  |  |  |
| A brain scan (MRI or PET) |  |  |  |  |
| Testing a new medicine |  |  |  |  |
| Travelling to hospital for study tests |  |  |  |  |

Q3. Have you been told by a doctor or nurse that you have any of the following? Please tick all the boxes that apply to you.

Some research studies have strict criteria for taking part. Your answers will help us understand how many people are prevented from taking part.

| Uncontrolled diabetes in the last 12 months |  |
| --- | --- |
| Uncontrolled high blood pressure in the last 12 months |  |
| Stroke or TIA (mini stroke) in the last 12 months |  |
| Serious heart disease (eg heart failure or recent heart attack) |  |
| Epilepsy with a seizure in the last 12 months |  |
| Severe kidney disease (very reduced kidney function) |  |
| Bleeding disorder *OR* take blood thinners (warfarin / Marevan® / Coumadin®, apixaban / Eliquis®, rivaroxaban / Xarelto®, edoxaban / Lixiana®, dabigatran / Pradaxa® |  |
| Taking high-dose steroids or medicines that suppress the immune system |  |
| A pacemaker or metal in your body |  |
| None of the above |  |
| Don’t know |  |
| Prefer not to say |  |

Q4. Sometimes practical things get in the way of taking part in research. Please tick.

|  | Yes | No |
| --- | --- | --- |
| MRI is not possible for me |  |  |
| I have significant hearing or vision problems that make tests hard |  |  |
| I don’t have anyone who could attend study visits with me |  |  |
| I would not be able to travel to the hospital |  |  |
| I would need travel or care costs reimbursed |  |  |

Q5: What would make it easier for you to take part in future research? Please write in the box below.

|  |
| --- |

Q5. I’d be happy to do simple brain-health checks **myself** at home; for example, an online memory test or a fingerprick blood sample.

| **Purpose of check** | **Strongly agree** | **Agree** | **Neutral** | **Disagree** | **Strongly disagree** | **Prefer not to say** |
| --- | --- | --- | --- | --- | --- | --- |
| To keep track of my brain health at home |  |  |  |  |  |  |
| To help doctors refer me to specialists sooner |  |  |  |  |  |  |
| To help match me to future research studies that would be a good fit for me |  |  |  |  |  |  |

Q6: What might put you off joining a dementia trial? Please tick all that apply.

| Too time consuming |  |
| --- | --- |
| Too far to travel |  |
| Don’t trust the researchers |  |
| Worried about safety or side-effects |  |
| Not sure what’s involved |  |
| Research materials do not reflect people like me |  |
| Research does not feel welcoming to me |  |
| Language barriers |  |
| Bad experience in the past |  |
| I’m not interested in a trial |  |
| Nothing would put me off |  |
| Other, please tell us | |

Q7: What would make you more likely to take part in dementia research?

| Knowing it could help future generations |  |
| --- | --- |
| Getting feedback about my own brain health |  |
| A video showing me what I would need to do |  |
| Flexible appointments |  |
| Visits at home |  |
| Being paid or getting travel expenses |  |
| Being able to ask questions and get support |  |
| Research that reflects people like me |  |
| A chance to try new treatments |  |
| I wouldn’t want to take part |  |
| Prefer not to say |  |
| Other, please state |  |

Q8: Which types of dementia research interest you most? Please tick your top 3.

| Preventing memory problems |  |
| --- | --- |
| Spotting memory problems earlier |  |
| Supporting carers and families |  |
| Finding new treatments and cures |  |
| Understanding causes of dementia |  |
| Improving daily life with dementia |  |
| Making care more personalised |  |
| I’m not interested |  |
| I don’t know |  |

**Section 4: Trust, Support and Impact**

Q1. If you were found to be at higher risk of developing dementia, would you trust NHS staff to explain what higher risk means and help you with next steps.  Please tick.

| Strongly agree | Agree | Neutral | Disagree | Strongly disagree | Don’t know | Prefer not to say |
| --- | --- | --- | --- | --- | --- | --- |
|  |  |  |  |  |  |  |

Q2. How did taking part in ACCESS D make you feel? Please tick all that apply.

| Reassured |  |
| --- | --- |
| Informed |  |
| It helped my mental health or sense of well-being |  |
| Curious to learn more |  |
| Keen to be involved in further stages of ACCESS D |  |
| Neutral |  |
| Confused |  |
| Frustrated |  |
| No effect |  |
| I found it stressful |  |
| Too soon to say |  |
| Prefer not to say |  |
| Other feeling: Please tell us here. | |

Q3. I trust dementia researchers to keep my data safe and use it in a positive way

| Strongly agree | Agree | Neutral | Disagree | Strongly disagree | Don’t know | Prefer not to say |
| --- | --- | --- | --- | --- | --- | --- |
|  |  |  |  |  |  |  |

Q4. Is dementia openly talked about in your family or community?

| Yes | No | I don’t know | Prefer not to say |
| --- | --- | --- | --- |
|  |  |  |  |
| Please tell us more here if you would like to. | | | |

Q5. Before you joined ACCESS D, was there anything that almost stopped you taking part?

| Yes, please tell us in the box |  |
| --- | --- |
| No |  |
| Prefer not to say |  |

Q6. The support from the ambulance staff made taking part easier

| Strongly agree | Agree | Neutral | Disagree | Strongly disagree | Don’t know | Prefer not to say |
| --- | --- | --- | --- | --- | --- | --- |
|  |  |  |  |  |  |  |

**Section 5: Some questions about you**

**These last questions ask a bit about you.**
Your answers help doctors and scientists understand how memory affects different people and communities. You can skip any questions you are not comfortable answering.

Q1. Please write your year of birth in the box.  For example, 1970

|  |
| --- |

Q2. Please tell us your gender?  We ask this because dementia is more common in females, and gender can affect people’s risk and experience of memory problems.

| Male |  |
| --- | --- |
| Female |  |
| Non-binary |  |
| Prefer not to say |  |

Q3. Please tell us your home postcode?

We won’t use your postcode to identify you. It will be turned into a score showing your local area and then deleted. This helps us check if the study is reaching all kinds of communities. If you would prefer not to answer, please skip this question.

|  |
| --- |

Q4. Please tick the option below that best describes your ethnicity.

We ask about your ethnicity because it helps us understand who is taking part in the ACCESS D study. This information is important to make sure that dementia research involves everyone and benefits people from all backgrounds.

| ASIAN: of Indian origin |  |
| --- | --- |
| ASIAN: of Pakistani origin |  |
| ASIAN: of Bangladeshi origin |  |
| ASIAN: of Chinese origin |  |
| ASIAN: of any other origin (please state) |  |
| BLACK: of African origin |  |
| BLACK: of Caribbean origin |  |
| BLACK: of other origin (please state) |  |
| MIXED: White and Black Caribbean |  |
| MIXED: White and Black African |  |
| MIXED: White and Asian |  |
| MIXED: Any other mixed or multiple ethnic background |  |
| WHITE: English, Welsh, Scottish, Northern Irish or British |  |
| WHITE: Irish |  |
| WHITE: Gypsy or Irish traveller |  |
| WHITE: Roma |  |
| WHITE: of any other origin |  |
| ARAB |  |
| ANY OTHER ETHNIC GROUP (please state) |  |
| Prefer not to say |  |
| Don’t know |  |

Q5. Is English your first language? Please tick.

| Yes | No | Prefer not to say |
| --- | --- | --- |
|  |  |  |

Q6. Please tell us the highest level of education you have completed.

We ask this question because understanding how memory problems and dementia affects people with different educational backgrounds helps scientists and doctors to develop fairer and more effective ways to support everyone.

| Primary school or equivalent |  |
| --- | --- |
| GCSEs or equivalent |  |
| A levels / Further education college / apprenticeship |  |
| University or higher |  |
| Prefer not to say |  |

Q7. How confident do you feel using smartphones, tablets, or computers? Please tick if you have one and how confident you feel using each one.

| Device | Please tick if you have one |
| --- | --- |
| Smart phone |  |
| Tablet |  |
| Computer |  |
| Reliable home internet |  |

| Device | How confident do you feel using these devices? | | | | |
| --- | --- | --- | --- | --- | --- |
|  | Very | Quite | Neither confident or unconfident | Not very confident | Not at all confident |
| Smartphone |  |  |  |  |  |
| Tablet |  |  |  |  |  |
| Computer |  |  |  |  |  |

**Thank you so much for taking the time to take part in ACCESS D.**

Your answers will help make research more fair, inclusive and meaningful for everyone.

Please use this space to tell us anything else, for example your thoughts about how to encourage more people to take part in dementia research from your community or anything else you would like us to know.

Your ideas could help shape future research in a way that really works for your community. Thank you.

| Best bit about ACCESS D:  What needs to change to make it better:  Suggestions to help more people from my community take part in research: |
| --- |

**With sincere thanks,**
The ACCESS D Team

If you have questions or would like to withdraw your details, please contact the ACCESS D team at p.a.fuller@soton.ac.uk.
