# Supplementary material for: Mixed-methods feasibility study of a community-based model to improve equity and efficiency in dementia research participation: protocol for ACCESS D
Source: BMJ Open. 2026 Jul 13;16(7):e118119. doi: 10.1136/bmjopen-2026-118119 (PMC13365783; doi:10.1136/bmjopen-2026-118119)
Supplement: Supplementary file 2 [file bmjopen-2026-118119.reviewer_comments.pdf]

# PEER REVIEW HISTORY

BMJ Open publishes all reviews undertaken for accepted manuscripts. Reviewers are asked to complete a checklist review form and are provided with free text boxes to elaborate on their assessment. These free text comments are reproduced below.

## ARTICLE DETAILS

**Title (Provisional)**

Protocol for ACCESS D: a mixed-methods feasibility study of a community-based model to improve equity and efficiency in dementia research participation

**Authors**

Fuller, Patricia; Claxton, Andrew; Pocock, Helen; Williams, Samantha Jane; Claxton, Nicola; Wollam, Amanda; Blackburn, Dan; Devitt, George; Fearn, Sarah; Kipps, Christopher

---

## VERSION 1 - REVIEW

---

| | |
|---|---|
| **Reviewer** | **1** |
| **Name** | **Sriram, Vimal** |
| **Affiliation** | **University Hospitals Bristol and Weston NHS Foundation Trust, Director of Allied Health Professionals** |
| **Date** | **27-Apr-2026** |
| **COI** | **None** |

---

Title of Manuscript: Protocol for ACCESS D: a mixed-methods feasibility study of a community-based model to improve equity and efficiency in dementia research participation.

I thank the editor for inviting me to peer-review this protocol manuscript. This is an important area of research and will benefit persons with dementia and researchers in dementia to co-produce and achieve equity in participatory research, especially those from underserved communities. This has likely implications and use in other specialities and long-term conditions.

A protocol manuscript needs to provide detailed process, procedures and rationale for what the research team intend to do, and so I have provided feedback on the manuscript which I hope the authors find useful to improve the manuscript. I have used page numbers and line references in the PDF version, to make it easier for authors to identify aspects that I am referring to.

1. Background and Rationale:

This section is structured well and the need for this research study is articulated.

a) Page 5 of 20 – Line 52 – The authors mention the Southcentral Ambulance Service – can they clarify here (or earlier in the manuscript) that this study is based in England, especially as the readership of this Journal is international and they may not be familiar with the geographical locations of the Team.

b) The research team is carrying out data collection in the southcentral region of England (Specifically Southampton and surrounding areas (Page 7- Lines 59,60) and as the study aims to evaluate inclusive access, it would be useful to have some additional information of the population within this region (compared with national indicators) – deprivation index, potential number of persons with dementia, education levels, ethnicity of the population, language profiles, access to digital devices/services, digital literacy/confidence etc.,

2. Methods and analysis:

The research objectives are well thought out and articulated.

a) Page 7 of 20 – The authors mention four work packages, but a timeline is only provided for work package 2, it will be useful to know timelines for the other work packages as well – fitting within their overall time frame of 12 months.

b) Page 7 of 20, Line 44 and also Page 13 of 20, Lines 20-24 – The authors mention using interviews and focus groups with participants conducted in person or by telephone – they have not mentioned here or elsewhere in the manuscript, how will they ensure that those with hearing impairment participate? Or for completion of questionnaires, those with visual impairment - Will there be reasonable adjustments for those who require it? If so, what would it be? Or will they be excluded – which defeats the purpose of the inclusive nature of this study.

b) Page 8 of 20 – Lines 4 and 5 – The authors mention likely settings – does this mean participants (inclusion criteria) are only those living in their own homes and can manage transport to one of the community locations by themselves – this would exclude those who cannot access transport or have physical accessibility issues or those in nursing/care homes etc., who could be the very underrepresented population this study should be targeting?

c) This study is targeted at under-represented population of people with dementia, I am assuming this would include people from black and minoritised ethnic backgrounds and those whose first language may not be English or may have poor literacy levels, or those with visual/auditory impairment etc., the authors should articulate if they have considered this and what alternate formats for providing information has been considered and where will they distribute this and how will they ensure this is in an accessible format?

3. Patient and public involvement and engagement:

The authors have included this as it is a necessary segment within this journal and have a person with lived experience as a co-author, which is great to see. However, more information is needed in this section, especially considering the inclusive nature of this study.

a) Page 9 of 20 - Line 3 – The authors mention that ACCESS D was developed with involvement from public contributors, it would be useful to know how many were involved, what was the nature of involvement and what were their backgrounds – i.e. are they from underrepresented communities?

4. Outreach events and implementation:

a) Page 10, Figure 2 – The authors mention elsewhere (Page 11, Line 37) that there are paper and digital versions for consent and presumably to access resources for this study, however Figure 2 only mentions QR codes to access Participant Information Sheets to then provide informed consent – this is likely to prevent those with digital poverty (no smart phones, no phones with cameras, no/limited data allowance to access material etc.,) or digital literacy issues in active participation/engagement with the study.

5. Participants:

a) page 11 of 20 - Eligibility criteria – Eligibility criteria is only listed for patient participants, however data is also being collected from Staff (Work package 3 – reflective interviews with SCAS staff; also Page 13 of 20 Lines 44-49 – debrief discussions, event evaluation logs and semi-structured interviews; also Page 15 of 20 Lines 49-50 – approximately 5 SCAS staff will be purposively sampled to ensure diversity and analytical adequacy), the authors should detail, who would be these staff members and what criteria is applied for their inclusion in this study?

6. Study procedures and data collection:

a) Table 2 mentions a 6 month follow up email, my earlier comments on digital poverty and digital literacy will apply here as well, and as this study is aimed at equity and representativeness, the authors should articulate what alternatives they will use to collect this information if not in a digital (email) format?

7. Optional research participation opportunities:

a) Page 13 of 20 Line 17 – The authors mention finger-prick blood sample will be collected for dementia biomarkers, however they also mention in Page 17 of 20 Lines 11 and 12 that the activities are non-diagnostic. I am unsure from this, what the research team would do with the blood samples, that are processed in the lab and how if something that needs to be communicated with the patient or their healthcare provider (GP for instance) will be done?

8. General comments:

a) Page 4 of 20 – Line 27 – the authors mention output of an implementation toolkit being an output from this work, however there is no mention of this in the manuscript on how this will be created, what it would potentially look like and who this will be aimed at.

b) Page 12 of 20 - The authors mention core activity questionnaire and provide broad areas that will be explored, I am unsure if this questionnaire is in development or is already available and if a sample can be provided as a supplementary file.

c) Page 15 of 20 – Data analysis - The authors need to clarify how they will deal with missing data in surveys, those lost to follow-up (especially as dementia is a progressive illness), how they will deal with establishing relations and rapport with the participants, especially highlighting who the interviewers/focus group facilitators are and – if the interviewer/facilitator is/are someone separate from the research team.

d) Page 16 of 20 – Qualitative analysis - the authors refer to inductive thematic analysis, however they do not mention who will be coding, their experience and expertise in qualitative research, how the data is being triangulated, how are they dealing with personal bias and reflexivity and details of their credentials in qualitative analysis and mixed-methods analysis.

e) There is a mention of Braun and Clark's six-phase approach (Page 16, Line 24) – this is not referenced in the list of references and similarly the authors need to reference the convergent mixed-methods design they are planning to use.

9. Discussion:

This section is adequate and written in the format for a protocol study.

---

## VERSION 1 - AUTHOR RESPONSE

Response to Reviewer 1

This section is structured well and the need for this research study is articulated.
Authors' response:
Thank you for this positive assessment of our manuscript.

Page 5 of 20 – Line 52 – The authors mention the Southcentral Ambulance Service – can they clarify here (or earlier in the manuscript) that this study is based in England, especially as the readership of this Journal is international and they may not be familiar with the geographical locations of the Team.
Authors' response:
Thank you for this helpful suggestion. We have revised the manuscript to add this clarification to the Abstract Introduction

'ACCESS D (Advancing Community Collaboration and Engagement Strategies in Dementia) aims to address these challenges by testing a community-based model that combines co-produced engagement events, low-burden research participation, and real-time support from

the South Central Ambulance Service (SCAS), a trusted, community-visible NHS healthcare workforce serving the counties of Hampshire, Oxfordshire, Buckinghamshire, and Berkshire in Southern England, UK.'

The research team is carrying out data collection in the southcentral region of England, specifically Southampton and surrounding areas, and as the study aims to evaluate inclusive access, it would be useful to have some additional information on the population within this region compared with national indicators: deprivation index, potential number of persons with dementia, education levels, ethnicity, language profiles, access to digital devices/services, digital literacy/confidence etc.
Authors' response:
We agree that this contextual information is important for interpreting the inclusive aims of the study. We have expanded the Setting section as below:

"ACCESS D will be delivered across non-clinical community venues in Southampton and surrounding areas. Southampton had an estimated resident population of 259,424 in 2024, with 13.7% aged 65 years and over, lower than the England average of 18.6%. The city is relatively deprived, ranking 76th most deprived of 296 English local authorities in 2025, and is ethnically and linguistically diverse: 31.9% of residents identify as other than White British, 15.4% do not have English as their main language, and nearly 160 languages are spoken locally.
Census 2021 data indicate a mixed educational profile in Southampton. Among usual residents aged 16 years and over, 17.3% had no formal qualifications, 9.5% had Level 1 or entry-level qualifications, 12.6% had Level 2 qualifications, 5.4% had an apprenticeship as their highest qualification, 20.5% had Level 3 qualifications and 31.6% had Level 4 or above qualifications. In Census 2021, Level 1 broadly corresponds to one to four GCSE passes or equivalent, Level 2 to five or more GCSE passes or equivalent, Level 3 to two or more A levels or equivalent, and Level 4 or above to higher education qualifications such as HNC/HND, degree, postgraduate or professional qualifications. These figures are broadly similar to the national profile for England, although Southampton has a slightly higher proportion with Level 3 qualifications and a slightly lower proportion with Level 4 or above qualifications than England overall.
Local dementia need is substantial: in 2025, 1,917 people aged 65 years and over had a recorded dementia diagnosis, while the estimated number living with dementia was higher at 2,480, projected to rise to 3,581 by 2045. Although local digital literacy estimates are limited, digital inclusion is identified as a local strategic priority, and national data suggest that 15% of UK adults lack foundation-level essential digital skills and 3% are offline."

The research objectives are well thought out and articulated.
Authors' response:
Thank you for this positive comment.

Page 7 of 20 – The authors mention four work packages, but a timeline is only provided for work package 2. It will be useful to know timelines for the other work packages as well, fitting within their overall time frame of 12 months.

Authors' response:
Thank you for this comment. We have revised the Methods section to provide start and end dates for each work package and to clarify how these fit within the overall 12-month project period.
Ethics, data-sharing, contracting and study set-up took place between September 2025 and February 2026.
WP1: Co-produce a community-based engagement model
'This work package (February to April 2026) involves co-developing…'
WP2: Implement and refine the outreach model
A minimum of 12–18 dementia-focused community outreach events will be delivered over six months (from February to July 2026).
WP3: Evaluate feasibility, acceptability and value
This WP will occur over 6 months (from February to July 2026) in parallel with WP2…
WP4: Evaluate progression and diversity across the participation pathway
The final work package (June to September 2026)…

Page 7 of 20, Line 44 and Page 13 of 20, Lines 20–24 – The authors mention interviews and focus groups with participants conducted in person or by telephone. They have not mentioned how they will ensure that those with hearing impairment participate, or how those with visual impairment will complete questionnaires. Will there be reasonable adjustments, or will they be excluded?
Authors' response:
Thank you for this comment. We agree that this required clearer description, particularly given the inclusive aims of the study. We have created a new section in Methods called Enhancing Inclusivity and have included the following paragraphs:

"Reasonable adjustments will be made wherever feasible. For participants with visual impairment, support may include large-print materials, paper copies, verbal administration of questionnaires and researcher-supported completion. For participants with hearing impairment, interviews and discussions may be conducted face-to-face in quiet spaces, with written prompts or written responses, video calls with captions where feasible, or support from a family member or carer if requested by the participant. Quiet spaces will also be available for participants who require a lower-stimulation environment."
"Participants will not be excluded solely because of hearing, visual, mobility, language or digital access needs, provided informed consent can be obtained and study activities can be completed safely and meaningfully with reasonable adjustments."

Page 8 of 20 – Lines 4 and 5 – The authors mention likely settings. Does this mean participants are only those living in their own homes and able to manage transport to community locations by themselves? This would exclude those with transport or physical accessibility issues, or those in nursing/care homes.
Authors' response:
Thank you for raising this important point. We have added the following to the Settings section:

"Venues will be selected co-productively with public contributors, community partners,

research paramedics and nurses and the academic team to maximise accessibility, cultural relevance and inclusion. Likely settings include community centres, places of worship, libraries, public events and other community-facing locations, including care homes and older people's residential complexes, to provide access for people with lower mobility and limited access to transport."

This study is targeted at under-represented populations of people with dementia. The authors should articulate whether they have considered people from Black and minoritised ethnic backgrounds, those whose first language may not be English, those with poor literacy levels, and those with visual/auditory impairment, and what alternate formats for information have been considered.
Authors' response:
Thank you. We agree and have expanded the Methods section to include a new section on Enhancing Inclusivity, which includes the following paragraphs:

"Participant-facing materials have been reviewed by PPIEP contributors and written in plain English. Summary and detailed participant information sheets will be available, with paper and large-print versions provided on request. At outreach events, SCAS research staff will provide real-time support, including explaining study activities, helping participants navigate digital tools, supporting questionnaire completion and answering questions."
"Events will be delivered in English, but NHS-approved dementia information leaflets in multiple languages will be available. For participants whose first language is not English, research staff will explain the study in simple terms and may involve family members or supporters where requested by the participant. Due to budget constraints, formal translation or interpreting services cannot be provided. Where language barriers mean that informed consent cannot be established, individuals will not be enrolled in the research component; however, they may still access general dementia information and signposting at the event. Unmet language needs that limit participation will be documented to inform future phases."

The authors have included this as it is a necessary segment within this journal and have a person with lived experience as a co-author, which is great to see. However, more information is needed in this section, especially considering the inclusive nature of this study.
Authors' response:
Thank you for this positive and helpful comment.

Page 9 of 20 – Line 3 – The authors mention that ACCESS D was developed with involvement from public contributors. It would be useful to know how many were involved, the nature of involvement, and their backgrounds, including whether they are from underrepresented communities.
Authors' response:
Thank you. We have expanded this section to describe the nature and contribution of public involvement in more detail.

"ACCESS D has been informed by previous public engagement and community dialogue work. Deliberative dialogue workshops conducted as part of a previous research project

involved a purposive sample of 50 members of the public from diverse communities and highlighted limited public understanding of what research involves and what researchers actually do. Community outreach conducted by the South Central Ambulance Service research team across a range of local groups and communities, including engagement with the local Vedic Hindu temple, also revealed strong support for bringing research into familiar community settings and providing opportunities for face-to-face discussion in a supported, low-pressure environment.
Two public contributors reviewed and informed participant-facing documentation. One contributor was of mixed Asian ethnicity with personal experience of dementia and extensive experience of community engagement with local ethnic minority communities. The second contributor was of White British ethnicity with personal and professional experience of dementia. Their input informed the use of plain English materials, summary and detailed participant information sheets, accessibility considerations, and community-based delivery. Public contributors will continue to contribute throughout the study through involvement in interpretation of findings, refinement of outreach approaches and dissemination activities, including co-produced lay summaries, presentations and peer-reviewed publications.

Page 10, Figure 2 – The authors mention elsewhere that there are paper and digital versions for consent and presumably access to study resources, but Figure 2 only mentions QR codes. This may prevent those with digital poverty or digital literacy issues from engaging.
Authors' response:
Thank you for identifying this inconsistency. We have revised Figure 2 to show that paper or digital options are available.

Eligibility criteria are only listed for patient participants, but data are also being collected from staff. The authors should detail who these staff members are and what criteria apply for their inclusion.
Authors' response:
Thank you for this comment. We have added staff eligibility criteria to the Participants section.

"Staff eligible for interviews or focus groups will include SCAS research paramedics, nurses and other delivery staff directly involved in planning, delivering or supporting ACCESS D outreach events or study procedures. Staff must be aged 18 years or over, able to provide informed consent and have sufficient experience of study delivery to contribute reflective feedback. Staff not directly involved in ACCESS D delivery will not be eligible. A purposive sample of approximately five staff members will be invited to ensure a range of delivery perspectives and experiences."

Table 2 mentions a 6-month follow-up email. Earlier comments on digital poverty and digital literacy also apply here. What alternatives will be used to collect this information if not by email?
Authors' response:
Thank you for spotting this. We agree and have revised the manuscript to clarify that follow-up will not be limited to email where this would exclude participants so this matches the questionnaire item allowing participants to provide their preferred mode of contact.

"Participants may opt to receive a brief follow-up communication via their preferred route, for example email, post, telephone or text message, approximately six months after participation, asking whether they have taken part in other dementia research since ACCESS D."

Page 13 of 20 Line 17 – The authors mention that a finger-prick blood sample will be collected for dementia biomarkers, but also state that the activities are non-diagnostic. What will the research team do with the blood samples, and how will anything that needs to be communicated to the patient or healthcare provider be handled?
Authors' response:
Thank you for raising this important point. We have revised the manuscript to clarify that finger-prick blood sampling is optional, exploratory and research-only and is not intended to provide a diagnosis, determine individual risk, or guide clinical care.

"Finger-prick blood sampling is included primarily to assess the feasibility and acceptability of collecting these blood samples in community settings, rather than to evaluate biomarker performance or return individual results. Samples will be used for exploratory measurement of biomarkers relevant to dementia research, such as amyloid, tau and/or neurodegeneration-related markers, subject to sample volume and assay feasibility.
Analyses will be conducted at group level only to explore feasibility, sample quality and the potential of ACCESS D to increase diversity of trial participation in the absence of an actively recruiting trial during the study period. Results will not be used for diagnosis, clinical decision-making or individual eligibility assessment, and individual results will not be returned to participants, as clearly detailed in participant information sheets."

We have also added the following paragraph to explain the rationale behind the research opportunities offered:
These activities were selected because they map onto issues that matter to people affected by memory concerns. Long waits for memory assessment and diagnosis can create uncertainty and anxiety; the 2023/24 National Audit of Dementia reported an average wait of 151 days from referral to diagnosis, ranging from 44 to 347 days across services.37 CognoMemory illustrates research aiming to support more efficient assessment pathways, while finger-prick sampling illustrates how less invasive approaches may, in future, support diagnosis and monitoring. In ACCESS D, both activities are exploratory, non-diagnostic and individual results will not be returned, but aim to demonstrate how worthwhile research can be.

Page 4 of 20 – Line 27 – The authors mention an implementation toolkit as an output, but there is no mention of how this will be created, what it would look like and who it will be aimed at.
Authors' response:
Thank you for this comment. We agree that the implementation toolkit required further explanation. We have added text describing the planned toolkit development process, intended audience and content.
"The toolkit will synthesise learning from co-production activities and is intended for

research teams, community partners and research delivery staff. It will include practical guidance on community engagement approaches, accessible communication, delivery considerations, reasonable adjustments, participant support, trust-building strategies, outreach planning, and approaches to improving inclusivity and pathway progression within dementia research participation."

Page 12 of 20 – The authors mention the core activity questionnaire and provide broad areas that will be explored. It is unclear whether this questionnaire is in development or already available and whether a sample can be provided as a supplementary file.
Authors' response:
Thank you for this comment. We have expanded the questionnaire section to describe how the ACCESS D questionnaire was developed and refined and it has now been included as a supplementary file.

'The core questionnaire was developed specifically for ACCESS D to capture feasibility, acceptability, barriers and enablers to dementia research participation, confidence and willingness to take part in future research, demographic characteristics and future contact preferences. Items were informed by the study aims, NIHR INCLUDE-aligned equity characteristics, prior public engagement work, PPIEP review and relevant literature on research participation and acceptability. The questionnaire includes a combination of Likert-scale and free-text items and incorporates the ECog-12 as a validated measure of everyday cognition. The questionnaire has been reviewed by public contributors for clarity, acceptability and accessibility and is included as a Supplementary File. As this is a feasibility study, the ACCESS D-specific items are not intended as a validated scale; their performance, completeness and acceptability will be assessed to inform refinement for future studies.'

Page 15 of 20 – Data analysis – The authors need to clarify how they will deal with missing data in surveys, those lost to follow-up, and how they will establish relationships and rapport with participants, including who the interviewers/focus group facilitators are and whether they are separate from the research team.
Authors' response:
Thank you. We have revised the Data analysis and qualitative methods sections to address these points.

"Missing data will not be imputed; patterns of missingness, non-completion, withdrawal and loss to follow-up will be described to inform the design, delivery and retention strategies of future studies."

'Interviews and focus groups will be conducted in person, online or by telephone according to participant preference and feasibility. Interviews, focus groups and qualitative coding will be conducted by PF, a medically trained mixed-methods member of the research team with a Master's degree in Public Health and three years' experience in qualitative and mixed-methods research. A subset of transcripts and coding will be reviewed by SF, a qualitative researcher in the research team with 17 years' experience, to support rigour. Rapport will be supported through plain-language explanations, flexible modes of participation, time for

questions, and sensitivity to participant preferences and support needs. At the start of interviews and focus groups, it will be emphasised that both positive and negative feedback are welcome and that there are no right or wrong answers. Reflexive notes will be maintained throughout data collection and analysis to support transparency regarding researcher perspectives and interpretation of findings.'

Page 16 of 20 – Qualitative analysis – The authors refer to inductive thematic analysis, but do not mention who will be coding, their experience and expertise, how data are being triangulated, how personal bias and reflexivity will be addressed, and details of credentials in qualitative and mixed-methods analysis.
Authors' response:
Thank you for this helpful comment. We have expanded the qualitative analysis section substantially.
'Interviews, focus groups and qualitative coding will be conducted by PF, a medically trained mixed-methods member of the research team with a Master's degree in Public Health and three years' experience in qualitative and mixed-methods research. A subset of transcripts and coding will be reviewed by SF, a qualitative researcher in the research team with 17 years' experience, to support rigour. Rapport will be supported through plain-language explanations, flexible modes of participation, time for questions, and sensitivity to participant preferences and support needs. At the start of interviews and focus groups, it will be emphasised that both positive and negative feedback are welcome and that there are no right or wrong answers. Reflexive notes will be maintained throughout data collection and analysis to support transparency regarding researcher perspectives and interpretation of findings.
Qualitative sampling will be purposive and iterative. As this is a feasibility study, recruitment will be guided by diversity of perspectives and data adequacy rather than formal theoretical saturation.39,40 The research team will monitor whether interviews and focus groups generate sufficient depth and breadth to address the study aims, including perspectives from underserved groups and those declining optional activities.
Mixed-methods integration
A convergent mixed-methods design will be used, whereby quantitative and qualitative data are collected during the same feasibility phase, analysed separately and then integrated during interpretation.41 Integration will use triangulation to compare areas of agreement, complementarity and divergence across quantitative findings, qualitative findings and implementation data, including event debriefs, delivery logs, field notes and staff reflections. A triangulation matrix will support systematic comparison across data sources to identify how different forms of evidence contribute to understanding feasibility, acceptability, equity, implementation challenges and optimisation of the ACCESS D model across different settings and participant groups.'

Braun and Clarke's six-phase approach is mentioned but not referenced. The authors also need to reference the convergent mixed-methods design.
Authors' response:
Thank you for noting these omissions. We have added the following references:
Braun V, Clarke V. Using thematic analysis in psychology. Qualitative Research in Psychology 2006;3(2):77–101.

Creswell JW, Plano Clark VL. Designing and Conducting Mixed Methods Research. 3rd ed. Thousand Oaks, CA: Sage; 2018.

This section is adequate and written in the format for a protocol study.
Authors' response:
Thank you for this positive comment.

---

**VERSION 2 - REVIEW**

---

| | |
|---|---|
| **Reviewer** | **1** |
| **Name** | **Sriram, Vimal** |
| **Affiliation** | **University Hospitals Bristol and Weston NHS Foundation Trust, Director of Allied Health Professionals** |
| **Date** | **16-Jun-2026** |
| **COI** | |

---

I thank the authors for accepting suggestions for revisions to their manuscript and for making comprehensive changes and indicating where the changes/additions have been made in the manuscript. I want to wish them well in the progress of this study and will look forward to reading the results from this work in the future.
